# Application of artificial intelligence in oral health management: challenges and opportunities

**DOI:** 10.3389/fmed.2026.1700529

**Published:** 2026-02-03

**Authors:** Hongcai Li, Shichao Chen, Bei Chang, Xinge Wang, Yuanpei He, Boya Xu, Guanyang Sun, Chaoyan Yang, Gang Li, Shiting Li, Guangwen Li

**Affiliations:** 1 Department of Stomatology, Shuguang Hospital Affiliated to Shanghai University of Traditional Chinese Medicine, Shanghai, China; 2 Luzhou Key Laboratory of Oral & Maxillofacial Reconstruction and Regeneration, The Affiliated Stomatological Hospital, Southwest Medical University, Luzhou, China; 3 Institute of Stomatology, Southwest Medical University, Luzhou, China; 4 Department of Stomatology, The PLA Rocket Force Characteristic Medical Center, Beijing, China; 5 Department of Stomatology, Norman Bethune International Peace Hospital, Shijiazhuang, China; 6 State Key Laboratory of Military Stomatology & National Clinical Research Center for Oral Diseases & Shaanxi Key Laboratory of Oral Diseases, School of Stomatology, The Fourth Military Medical University, Xi’an, China; 7 Department of Stomatology, The 941 Hospital of the Joint Service Support Force of the People’s Liberation Army of China, Xining, China; 8 Department of Stomatology, The 83 Affiliated Hospital of Xinxiang Medical University, Xinxiang, China; 9 Department of Pediatrics, Luzhou People’s Hospital, Luzhou, China; 10 Faculty of Stomatology, College of Medicine, North West University, Xi’an, China

**Keywords:** artificial intelligence, oral health data, oral health education and counseling, oral health management, personalized treatments, tele-dentistry

## Abstract

**Objectives:**

Artificial intelligence (AI) is increasingly being utilized across various fields of medicine, presenting significant potential for the future of healthcare. This review is to systematically outline the current applications of AI in the field of oral health management and to provide an in-depth analysis of the associated challenges and future opportunities.

**Methods:**

The review was based on a systematic electronic literature search conducted across databases (PubMed, Web of Science, and Scopus) with the keywords including “artificial intelligence,” “AI in dentistry,” “tele-dentistry,” “oral health education,” and “oral health management.” English-language studies relevant to the application of AI across various aspects of oral health management were included based on independent assessments by two reviewers.

**Results:**

We concluded that in the realm of oral health management, AI technology has diverse applications, including oral health education and counseling, monitoring, screening, diagnosis, treatment, follow-up care of oral diseases, and the collection and management of oral health data. By enhancing public awareness of oral health and improving self-management capabilities, AI can increase diagnostic accuracy, facilitate personalized treatments, support tele-dentistry, optimize the allocation of dental resources, and provide early warnings for oral diseases. These advancements collectively contribute to the efficiency and quality of oral health management. While AI demonstrates considerable promise in this field, several challenges remain, including inconsistencies in oral health data, limited availability and accessibility of data, the reliability of AI-driven results, and issues of bias and fairness in AI algorithms. Addressing these challenges is essential to fully harness the transformative potential of AI in oral health management.

**Conclusion:**

Oral health management encompasses the comprehensive handling of oral health risk factors in individuals, populations, and communities through a series of measures and activities aimed at maintaining and promoting oral health. The ultimate goal is to achieve the greatest societal benefit in oral health at the lowest possible cost. By addressing challenges such as data consistency, availability, and reliability, as well as issues of bias and fairness in AI algorithms, AI may play a significant role in oral health management.

**Clinical relevance:**

This paper reviews the role of artificial intelligence in the prevention, diagnosis and treatment of oral diseases, providing an important reference for the later application of artificial intelligence in oral health management.

## Introduction

Oral diseases, including dental caries, periodontal disease, tooth loss, oral mucosal lesions, and oropharyngeal cancer, represent significant public health challenges worldwide, imposing considerable burdens on both health systems and economies (1–3). Poor oral health has extensive repercussions for overall health and quality of life. Fortunately, oral diseases are predominantly preventable and can be addressed through the implementation of effective preventive measures and the promotion of oral health. Effective oral healthcare necessitates the integration of clinical and preventive dental services, with an increased emphasis on promoting and sustaining oral health, as well as achieving greater equity in oral health outcomes (3). Oral health management encompasses the comprehensive management of oral health risk factors for individuals, populations, and communities through a series of measures and activities aimed at maintaining and enhancing oral health. This management involves not only routine oral care but also prevention, diagnosis, treatment, and health education, among other components. The fundamental strategy of oral health management is to maximize public oral health benefits while minimizing costs.

**FIGURE 1:**
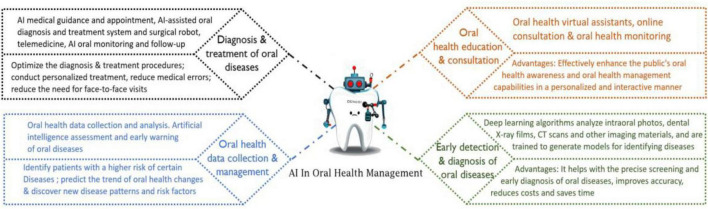
The applications of artificial intelligence in various aspects of oral health management.

The emergence of artificial intelligence (AI) can be traced back to John McCarthy’s pioneering articulation of the AI concept, which has undergone remarkable advancements since its inception (4, 5). The widespread application of AI in the healthcare industry has accelerated significant progress in areas such as intelligent diagnosis (6), medical image recognition (7), medical robotics (8), smart drug development (9), and intelligent health management (10). Moreover, AI is being utilized across various medical fields, presenting tremendous potential for the future of healthcare (11–13). By offering personalized patient experiences, improving treatment outcomes, facilitating early diagnosis, enhancing clinicians’ capabilities, increasing operational efficiency, and improving the accessibility of healthcare services, AI provides healthcare institutions with a competitive edge (14–16). Furthermore, the application of AI in healthcare has the potential to balance the distribution of medical resources, ensuring that everyone, regardless of location or economic status, can access high-quality healthcare (17–19). It is foreseeable that, with the support of AI technology, the era of smart healthcare is on the horizon. The integration of AI in oral medicine has led to the development of innovative models that significantly enhance oral healthcare (20–23). The application of AI in oral health management is revolutionizing traditional healthcare paradigms by enhancing diagnostic accuracy, personalizing treatment, optimizing healthcare resources, and improving patient education. These advancements collectively not only promote the efficiency and quality of oral health management but also lay the foundation for a more comprehensive and effective public health system. Given the multifaceted systemic interactions in oral health management, the development and application of AI in this field remain in the early stages. This article examines the current applications of AI technology in various facets of oral health management, encompassing oral health education, monitoring, screening, diagnosis, treatment, follow-up care, and the collection and management of oral health data (Figure 1). It also addresses the challenges and limitations that must be confronted. The objective is to offer a comprehensive overview of the impact of AI on oral health management and to pinpoint areas that necessitate further development and attention.

## Methods

We screened original research articles published since 2019 on the applications of artificial intelligence (AI) in various aspects of oral health management across three electronic databases (PubMed, Scopus, Web of Science). The research questions were formulated using the acronym PCC:

1. Participants (P): Studies involving human oral health or diseases, or those focusing on oral medical imaging and data.
2. Concept (C): Studies centered on the development, evaluation, or application of artificial intelligence/machine learning technologies.
3. Context (C): Application scenarios covering any aspect of oral health, including but not limited to disease diagnosis, treatment planning, prognosis prediction, patient management, public health, and medical education.

### Search strategy and article selection

A systematic search strategy was implemented for each database, as presented in Table 1. The inclusion criteria applied to original research articles, while the exclusion criteria comprised case reports, technical reports, narrative articles, conference papers, scoping reviews, and systematic reviews. Additionally, non-English literature, studies with inaccessible full texts, studies involving animals, and literature focusing solely on simple automation or computerization without core machine learning technologies were excluded.

**TABLE 1:** The keywords of an electronic search in various databases.

Database	Keywords
PubMed	#1 “Artificial Intelligence”[Mesh] OR “Machine Learning”[Mesh] OR “Deep Learning”[Mesh] OR “Neural Networks, Computer”[Mesh] OR artificial intelligence[tiab] OR machine learning[tiab] OR deep learning[tiab] OR neural network*[tiab] OR computer vision[tiab] OR AI[tiab] #2 “Dentistry”[Mesh] OR “Oral Health”[Mesh] OR “Dental Care”[Mesh] OR “Tooth”[Mesh] OR “Mouth”[Mesh] OR dentistr*[tiab] OR dental[tiab] OR odont*[tiab] OR “oral health”[tiab] OR “oral medicine”[tiab] OR “oral disease*”[tiab] OR tooth[tiab] OR teeth[tiab] OR periodont*[tiab] OR orthodont*[tiab] OR endodont*[tiab] OR “oral cancer”[tiab] OR “oral carcinoma”[tiab] OR “mouth neoplasms”[tiab] OR caries[tiab] OR cavity[tiab] OR “dental implant*”[tiab] #3 #1 AND #2
Scopus	(TITLE-ABS-KEY (“artificial intelligence” OR “machine learning” OR “deep learning” OR “neural network*” OR “convolutional neural network” OR cnn OR “computer vision” OR “natural language processing” OR nlp OR “random forest” OR “support vector machine” OR svm OR “transfer learning”) AND NOT TITLE-ABS-KEY (“artificial insemination” OR “academic integrity” OR “autoimmune”) AND (TITLE-ABS-KEY (dentist* OR dental OR “oral health” OR “oral medicine” OR “oral disease*” OR tooth OR teeth OR periodont* OR orthodont* OR endodont* OR “oral cancer” OR “oral carcinoma” OR “mouth neoplasm*” OR caries OR “dental caries” OR cavity OR “dental implant*” OR maxillofacial OR “temporomandibular joint” OR tmj OR saliva OR “oral microbiome” OR “digital dentistry” OR “intraoral scan*” OR cbct OR “cone beam”)
Web of Science	#1: TI = [(“artificial intelligence” OR “machine learning” OR “deep learning” OR “neural network*” OR “convolutional neural network” OR “CNN” OR “transfer learning” OR “supervised learning” OR “unsupervised learning” OR “computer vision” OR “natural language processing” OR “NLP” OR “random forest” OR “support vector machine” OR “SVM”) NOT (“immune” OR “immunity” OR “immune system”)] #2: dentist*” OR “dental” OR “oral health” OR “oral medicine” OR “oral disease*” OR “tooth” OR “teeth” OR “periodont*” OR “orthodont*” OR “endodont*” OR “oral cancer” OR “oral carcinoma” OR “mouth neoplasm*” OR “caries” OR “dental caries” OR “cavity” OR “dental implant*” OR “maxillofacial” OR “temporomandibular joint” OR “TMJ” OR “saliva” OR “oral microbiome” #3 #1 AND #2

The literature screening process was conducted independently by two reviewers and cross-checked thereafter. Any discrepancies between the two reviewers were resolved through discussion following consultation with a third expert. The process is illustrated in the figure below and adheres to the PRISMA flow diagram.

## Results

### Study selection

**FIGURE 2:**
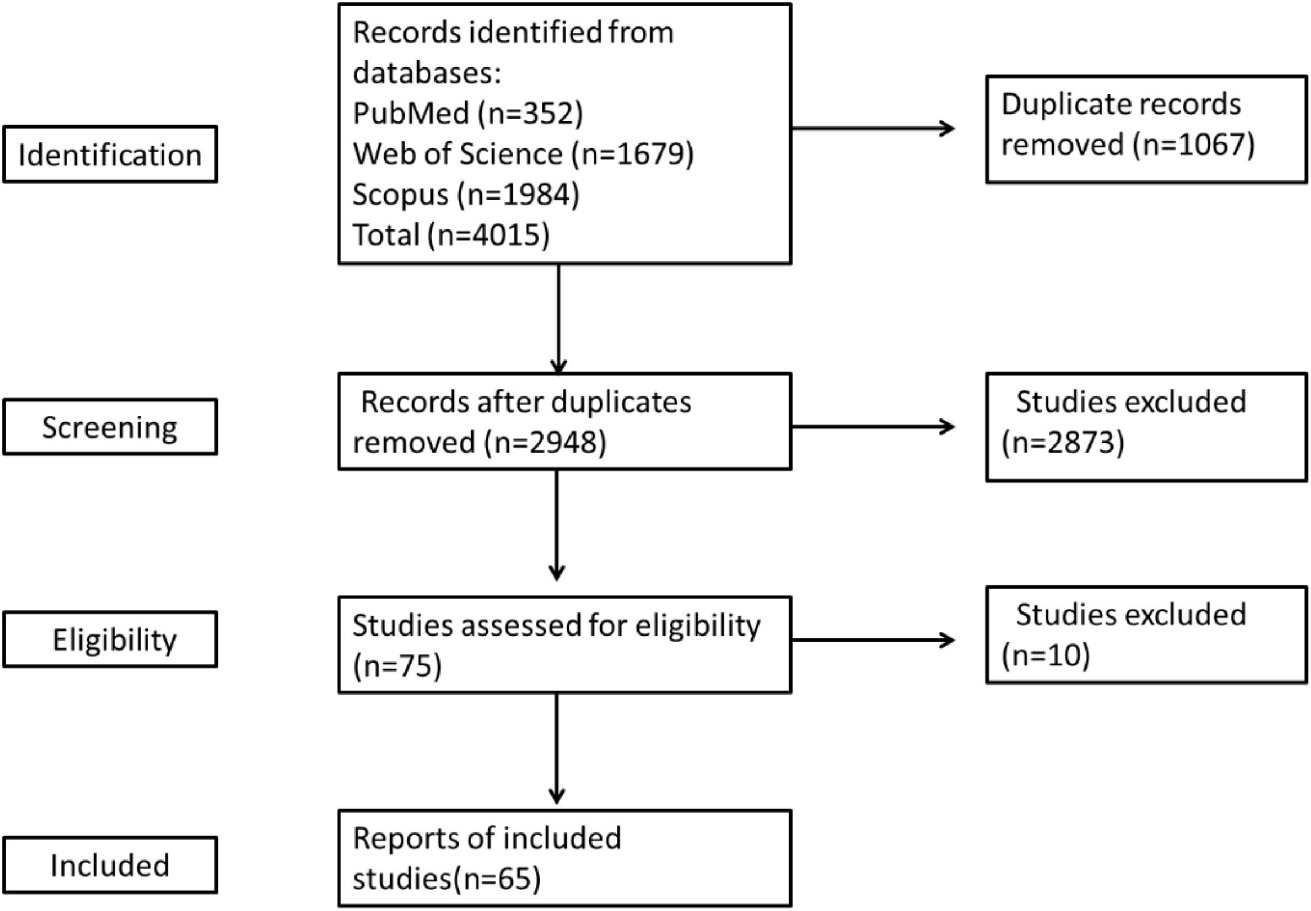
PRISMA flow diagram illustrating the search process.

The systematic search retrieved 2,948 unique records. After screening the titles and abstracts, 2,873 records were excluded. Of the remaining 75 records, 10 were excluded after full-text review. A total of 65 articles were finally included in this study. The detailed study selection process is summarized in the PRISMA flowchart shown in Figure 2.

Table 2 outlines the characteristics of the 65 studies included in this analysis. These studies explored the potential applications of AI in various aspects of oral health management, including oral health education and counseling, monitoring, screening, diagnosis, treatment, follow-up care for oral diseases, as well as the collection and management of oral health data.

**TABLE 2:** Characteristics of the included studies.

Year	References	Objective of the study	AI model or devices	Algorithm architecture	Study factor	Modality	Application fields	key findings or conclusions	study type
2024	Lv et al. (24)	To assess the effectiveness in offering recommendations for common oral health issues	Google Bard, ChatGPT-3.5, ChatGPT-4	LLMs	Responses to the questions	Questions on prevalent oral diseases	Oral health education	LLMs, particularly ChatGPT-4, providing patient-centric information for enhancing patient education and clinical care.	Observational Study
2024	Shafaee et al. (25)	To compare the effectiveness on periodontal health in fixed orthodontic patients	WhatsApp	An android application	PI, GI	Fixed orthodontic patients	Oral health education	The specially designed software could be the most effective for improving oral hygiene in orthodontic patients.	Observational Study
2022	Wu et al. (26)	To evaluate the effectiveness on oral hygiene promotion	Clean Teet	A WeChat mini-program	PI, GI	Fixed orthodontic patients	Oral health education	The HAPA theory-based mini-program had positive effects on oral-health behavior promotion and oral hygiene among young adults with fixed orthodontic appliances.	Experimental Study
2021	Zolfaghari et al.(27)	To design an app and assess its efficacy for education of mothers regarding oral healthcare of their children	A simple app and a gamified app	A smartphone application	A questionnaire of mothers, PI of children	Mothers of preschoolers	Oral health education	Both apps effectively improved the oral-health knowledge and practice of mothers while oral hygiene as a result of plaque control was superior in children of mothers using the gamified app.	Experimental Study
2025	Stephan et al.(28)	To evaluate effectiveness on patient understanding, readability, and communication quality	ChatGPT	LLMs	Text analysis, Readability Indices	Radiology reports	Health care communication	AI-generated simplification of radiology reports significantly enhances patient comprehension and engagement.	Comparative study
2024	Hunsrisakhun et al.(29)	To compare the effectiveness for caregivers in improving young children’s oral health	The 21-Day FunDee Plus	Chatbot	A questionnaire, Caries, plaque	Caregiver–child pairs (aged 6–42 months)	Oral health education	The standalone chatbot 21-Day FunDee Plus presents a viable alternative for promoting oral health in young children.	Comparative study
2022	Pithpornchaiyakul et al.(30)	To evaluate the effectiveness and usability of 2 chatbots	21-Day FunDee and 30-Day FunDee	Chatbot	A questionnaire, plaque	Caregivers for children (aged 6–36 months)	Oral health education	Both chatbots significantly improved overall knowledge, overall oral health care perceptions, and toothbrushing for children by caregivers.	Observational Study
2025	Chen et al.(31)	To compare orthodontic pre-treatment information provided	Ernie Bot, ChatGPT, and Gemini	LLMs	Responses to the questions	Pre-treatment consultation questions	Oral health consultation	Scores for each group in various dimensions primarily fell within the range of 3–4 points, with relatively few high-quality scores (5 points).	Comparative study
2025	Özcivelek et al. (32)	To evaluate the accuracy, quality, readability, understandability, and actionability of the responses	DeepSeek-R1, ChatGPT-o1, ChatGPT-4, and Dental GPT chatbots	Chatbots	Responses to the questions	Questions about prostheses	Oral health education	While accuracy may vary among chatbots, the domain-specific trained AI tool and ChatGPT-o1 demonstrated superior accuracy.	Comparative study
2025	Santonocito et al. (33)	To evaluate the impact on orthodontic patient education	AI-based chatbots	Chatbots	PI, MGI, a questionnaire	Orthodontic patients	Oral health education	The use of AI-based chatbots positively influences orthodontic oral hygiene in orthodontic patients.	Observational Study
2025	Tuzlalı et al. (34)	To evaluate and compare the performance in providing responses to frequently asked questions (FAQs) about dental implant treatment.	ChatGPT-o1, Deepseek-R1, Google-Gemini-Advanced, Claude-3.5-Sonnet, and Perplexity-Pro	LLMs	Responses to the questions	Questions regarding dental implants	Dental education and patient communication	AI-driven chatbots demonstrated strong potential in delivering accurate and patient-friendly information about dental implant treatment. However, performance varied considerably across platforms, with ChatGPT-o1 and Deepseek-R1 showing the highest reliability.	Comparative study
2024	Schwarzmaier et al. (35)	To evaluate the external validation for detecting ECC and classifying carious lesions in dental photographs.	A web tool	–	Caries	Dental photographs	Caries detection	ECC detection accuracy was 97.2%.	Experimental Study
2023	Deng et al. (36)	To develop a multiclass non-clinical screening tool for periodontal disease and assess its accuracy	A RF-based multiclass classifier	LR. and RF	Periodontal disease classification	A questionnaire, aMMP-8 POCT and GBoB.	Periodontitis, auxiliary diagnosis	Machine learning-based classifiers, such as RF analyses, are promising tools for multiclass assessment of periodontal health and disease in a non-clinical setting.	Observational Study
2024	Li et al. (37)	To assess the effect of AI-MST system as an adjunct to clinical practice guideline-conform treatment.	Oral-B IO9	Oral-B App	Inflamed periodontal pockets, gingival inflammation	Periodontitis adult patients	Oral health care	The tested digital health intervention significantly improved the outcome of periodontal therapy by enhancing the adherence and performance of self-performed oral hygiene.	Experimental Study
2024	Marashi et al. (38)	To assess the effect on the promotion of behaviors related to oral health	Teeth Care educational application	A mobile application	Questionnaires	Adolescents (aged 13–15 years)	Oral health educational	The educational intervention through the mobile application was effective in improving the average scores of awareness, attitude, self-efficacy in brushing teeth, flossing, visiting the dentist, and the behavior of adolescents regarding oral health behaviors.	Experimental Study
2025	Hu et al. (39)	To evaluate the effects on PM, behavioral change, and periodontal treatment outcomes.	DentalMonitoring app	A mobile application	PPD and CAL, a questionnaire	Periodontitis patients	Oral health monitoring	AI-assisted monitoring improved self-care behaviors, plaque control, and periodontal clinical outcomes.	Experimental Study
2025	Islam et al. (40)	To compare the effectiveness in improving patient comprehension and reducing anxiety	ChatGPT	LLMs	A questionnaire	Educational materials	Oral health education	AI-generated educational materials demonstrate superior effectiveness in improving patient comprehension and reducing anxiety compared to traditional methods.	Comparative study
2025	Jeong et al. (41)	To develop and evaluate a system that automatically detects and quantifies dental plaque	A DL model	Tensor Flow framework	Quigley-Hein plaque index	Dental images	Plaque assessment and remote dental	The DL-based system successfully automated the evaluation of dental plaque from images, performing comparably to an experienced clinician.	Experimental Study
2025	Chau et al. (42)	To evaluate the accuracy of a new mHealth tool in detecting gingivitis, and the user acceptance of personalized oral hygiene instructions provided through the new tool	GumAI	DeepLabv3 + machine learning architecture	Gingivitis	Intraoral photographs	mHealth systems, oral hygiene management	GumAI demonstrates high sensitivity, PPV, accuracy, and F1 score compared to the panel’s assessments but falls relatively short in specificity and NPV. Despite this, the tool was highly accepted by older adults.	Experimental Study
2025	Zhang et al. (43)	To establish a CRA platform for managing caries	CRA model	LR	Deciduous caries	Children (aged 3–5 years)	Oral health screening and management	The CRA model offers a novel public platform with the potential to serve as an effective tool for the screening and management of deciduous caries at the community level in underdeveloped regions of Northwest China.	Experimental Study
2024	Fanelli et al. (44)	To introduce a specialized AI model designed to provide up-to-date periodontal knowledge	PerioGPT	LLMs	Responses to the questions, evaluations of the generated questions	Periodontal questions, generate questions related to periodontology	Dental educational and clinical applications	PerioGPT outperformed other chatbots, achieving a higher accuracy rate (81.16%) and generating more complex and precise questions.	Experimental study
2025	Li et al. (45)	To compare the effectiveness for veneer tooth preparation training and evaluate the impact of training sequence on skill acquisition.	Unidental, simodont	VR	Marginal integrity, preparation depth, proximal contour, and surface smoothness	Veneer tooth preparation training	Dental education	The study found no significant differences in training outcomes between VR simulators and traditional phantom heads for veneer preparation.	Comparative study
2025	Huang et al. (46)	To evaluate the potential of ChatGPT in clinical operative skills education.	ChatGPT-3.5	LLMs	A knowledge test, operational test, purdue spatial visualization test, questionnaires	Undergraduate dental students	Dental education	ChatGPT has performed outstandingly in assisting dental skill learning, and the study supports the integration of ChatGPT into skills teaching and provides new ideas for modernizing skill teaching.	Experimental Study
2023	Weingart et al. (47)	To evaluate the accuracy for automated identification of 60 cephalometric landmarks	DNPs	DL	60 cephalometric landmarks	Skull CT	Cephalometric analysis	The DNP was successfully trained to identify all 60 landmarks.	Experimental study
2025	Pan et al. (48)	To validate the accuracy and reliability of a superimposition method	MDM	GCN	Tooth position and angulation	Dental casts scanning images	Orthodontic outcome evaluation	The GCN-based MDM superimposition is an efficient method for the assessment of tooth movement in adults.	Experimental study
2019	Vinayahalingam et al. (49)	To achieve an automated high-performance segmentation of the third molars, and the inferior alveolar nerves (IAN)	U-net	CNN	Inferior alveolar nerve (IAN) and lower third molars (M3)	Panoramic radiographs	Oral surgery, clinical decision making	Deep-learning is an encouraging approach to segment anatomical structures. Further enhancement of the algorithm is advised to improve the accuracy.	Experimental study
2019	Hiraiwa et al. (50)	To examine the diagnostic performance for classification of the root morphology of mandibular first molars	AlexNet, GoogleNet	DL	Root morphology of mandibular first molars	Panoramic radiographs	Auxiliary diagnosis	The deep learning system showed high accuracy in the differential diagnosis of a single or extra root in the distal roots of mandibular first molars.	Observational study
2019	Park et al. (51)	To compare the accuracy and computational efficiency for automatic identification of cephalometric landmarks.	YOLOv3, SSD	CNN	Cephalometric landmarks	Cephalometric radiographic images	Orthodontics	Between the two latest deep-learning methods applied, YOLOv3 seemed to be more promising as a fully automated cephalometric landmark identification system for use in clinical practice	Comparative study
2019	Kök et al. (52)	To determine CVS for growth and development periods by the frequently used seven artificial intelligence classifiers, and to compare the performance of these algorithms with each other.	k-NN, NB, Tree, ANN, SVM, RF, LR	ANN	CVS	Cephalometric radiographs	Determining growth development	k-NN and Log.Regr. algorithms had the lowest accuracy values. SVM-RF-Tree and NB algorithms had varying accuracy values. ANN could be the preferred method for determining CVS.	Comparative study
2024	Stephan et al. (53)	To assess the effectiveness in generating radiology reports from dental panoramic radiographs	ChatGPT	LLMs	Radiology reports	Panoramic radiographs	Dental radiology	Reports generated by ChatGPT showed a high degree of textual similarity to reference reports, but lacked critical diagnostic information typically included in reports authored by students.	Comparative study
2024	Szabó et al. (54)	To assess the reliability of AI-based system that assists the healthcare processes in the diagnosis of caries on intraoral radiographs	Diagnocat system	CNN	Carious	Intraoral bitewing and periapical radiographs	Auxiliary diagnosis	The Diagnocat CNN supports the evaluation of intraoral radiographs for caries diagnosis, as determined by consensus between human and AI system observers.	Experimental study
2019	Casalegno et al. (55)	To present a deep learning model for the automated detection and localization of dental lesions in near-infrared transillumination (TI) images	A DL model	CNN	Dental caries	TI imaging	Auxiliary diagnosis	The deep learning approach for the analysis of dental images holds promise for increasing the speed and accuracy of caries detection.	Experimental study
2019	Hung et al. (56)	To develop and evaluate a ML model to select the most relevant variables in classifying root caries	SVM, XGBoost, RF, k-NN, LR.	Machine learning	Dental caries	Public data	Guide treatment decisions	Of the machine learning algorithms developed, SVM demonstrated the best performance for identifying root caries. Age was the feature most strongly associated with root caries.	Experimental study
2019	Kim et al. (57)	To propose a DL-based method for developing an automated diagnostic support system that detects periodontal bone loss in the panoramic dental radiographs	DeNTNet	CNN	Periodontal bone loss	Panoramic radiographs	Auxiliary diagnosis	Through the multi-step training framework, the proposed model was able to achieve a PBL detection performance superior to that of dental clinicians.	Experimental study
2025	Do et al. (58)	To develop and validate an AI-driven system for automated staging and grading periodontitis from panoramic radiographs	AI-assisted periodontal diagnosis interface	YOLOv8 model and Gradio	Periodontitis staging and grading	Panoramic radiographs	Dental radiology, auxiliary diagnosis	The proposed YOLOv8-based framework accurately detects key periodontal landmarks and automates disease staging and grading	Experimental study
2019	Krois et al. (59)	To apply deep CNNs to detect periodontal bone loss (PBL) on panoramic dental radiographs	CNN	CNN	Periodontal bone loss (PBL)	Panoramic radiographs	Periodontitis, auxiliary diagnosis	A CNN trained on a limited amount of radiographic image segments showed at least similar discrimination ability as dentists for assessing PBL on panoramic radiographs.	Experimental study
2023	Altukroni et al. (60)	To design and evaluate an AI tool for detecting pathological exposure of pulp on digital periapical radiographs and to compare its performance with dentists.	Make sure caries detector and classifier (MSc)	Yolov5-x	Pathological exposure	Digital periapical radiographs	Dental radiology, auxiliary diagnosis	MSc is proved reliable in the detection and differentiating between exposed and unexposed pulp in the internally validated model. It also showed a better performance compared to the 10 dentists’ consensus.	Experimental study
2025	Liu et al. (61)	To utilize two DL models to aid novice dentists in the detection of periapical lesions (PLs) on periapical radiographs (PRs)	ConvNeXt, ResNet34	CNN	Periapical lesion	Periapical radiographs	Dental radiology, auxiliary diagnosis	ConvNeXt significantly improved the diagnostic performance of novice dentists and reduced the time required for diagnosis, thereby enhancing clinical efficiency in both diagnosis and treatment.	Comparative study
2024	Marya et al. (62)	To develop a deep learning model to predict skeletal malocclusions using airway and cephalometric landmark values	Tree, RF, Gradient Boosting, SVM, k-NN, LR, and ANN	Supervised learning	Skeletal malocclusion	CBCT	Orthodontics, auxiliary diagnosis	The RF model was the most accurate model for predicting the skeletal malocclusion based on various airway and cephalometric landmarks.	Experimental study
2019	Kise et al. (63)	To estimate the diagnostic performance of a deep learning system for detection of Sjögren’s syndrome (SjS) on CT, and compare it with the performance of radiologists.	AlexNet	DL	Sjögren’s syndrome (SjS)	CT	Oral mucosal diseases, auxiliary diagnosis	The deep learning system showed a high diagnostic performance for SjS, suggesting that it could possibly be used for diagnostic support when interpreting CT images.	Observational study
2020	Lee et al. (64)	To evaluate the discriminating performance on the classification of specific features of osteoporosis	CNN3, VGG-16, VGG-16_TF,VGG-16_TF_FT	CNN	Osteoporosis	Panoramic radiographs	Dental radiology, auxiliary diagnosis	DL -based assessment of DPR images could be useful and reliable in the automated screening of osteoporosis patients.	Comparative study
2025	Wang et al. (65)	To design and promote a novel approach for tooth segmentation, numbering and abnormal morphology detection in panoramic X-ray (PX)	Pxseg	DL	Tooth segmentation, abnormal teeth morphology detection	CBCT and panoramic radiographs	Dental radiology, auxiliary diagnosis	The application of accurate labels in ctPX increased the pre-training weight of PXseg and improved the training effect, achieving promotions in tooth segmentation, numbering and abnormal morphology detection.	Experimental study
2019	Men et al. (66)	To develop a xerostomia prediction model with radiation treatment data using a three-dimensional residual convolutional neural network (3D rCNN)	A 3D rCNN model	rCNN	Predicting xerostomia	CT, 3D dose distributions, contours of the parotid and submandibular glands	Supporting clinical decision-making.	The proposed model achieved promising prediction results.	Experimental study
2024	Yıldız et al. (67)	To predict temporomandibular disorder (TMD) using ML approaches based on measurement parameters	Bagging algorithm	MARS algorithm	Predict temporomandi- bular disorder (TMD)	The clinical measurement parameters	Auxiliary diagnosis	The ML algorithm proposed in this study may assist clinicians inexperienced in TMD to make a preliminary detection of TMD in clinics where diagnostic imaging tools are limited.	Experimental study
2025	Furquim et al. (68)	To construct predictive models of periodontitis progression	LR, multi-layer perceptron (MLP) and probabilistic graphic model (PGM)	ML	Predict periodontitis progression	Periodontal examination, levels of salivary analytes, clinical and demographic parameters	Supporting early detection strategies	The PGM model demonstrated the best overall performance compared to LR and MLP.	Experimental study
2024	Feng et al. (69)	To propose a data-driven AS decision support system, integrating both supervised and unsupervised ML for efficient patient grouping and scheduling	Cluster-Predict-Schedule (CPS)	ML	Generating appointment templates	Patients’ service time	Outpatient appointment scheduling	Our system’s efficacy is demonstrated using a real-world dataset.	Experimental study
2024	Elgarba et al. (70)	To assess quality, clinical acceptance, time-efficiency, and consistency of a AI-driven tool for automated presurgical implant planning for single tooth replacement	A cloud-based platform (Relu^®^ Creator, Belgium)	3D U-networks	Implant planning	CBCT, intra-oral scans (IOS)	Preoperative implant planning	AI demonstrated expert-quality and clinically acceptable single-implant planning, proving to be more time-efficient and consistent than the HI-based approach.	Experimental study
2025	Roongruangsilp et al. (71)	To evaluate the performance of two object detection models in analyzing cross-sectional and panoramic images derived from DICOM files processed by four distinct dental imaging software platforms.	Faster R-CNN and YOLOv7	CNN	Implant position images	CBCT	Dental implant planning	Faster R-CNN achieved superior accuracy across imaging modalities, while YOLOv7 demonstrated higher detection rates, albeit with lower precision.	Comparative study
2025	Binvignat et al. (72)	To compare the performances of different AI approaches for the learning and reconstruction of central incisors.	Principal component analysis (PCA) and DL of Signed Distance Functions (DeepSDF)	Feature contribution analysis, Stochastic Neighbor Embedding (t-SNE)	Learning and reconstruction of central incisors	Mature permanent maxillary incisors	Smile designs and oral rehabilitation	DeepSDF showed significantly better precision in surface, volume, and Hausdorff distance metrics compared with PCA. For reconstructions, the lower size of the latent code of the DeepSDF model demonstrated lower performances compared with higher sizes.	Comparative study
2025	Wu et al. (73)	To evaluate the time efficiency and morphological accuracy of designed crowns	AI Automate (AA) and AI Dentbird Crown (AD)	GANs, CNN	Crown design	Intraoral scan datasets	Dental restoration	AI-powered software enhanced the efficiency of crown design but failed to excel at morphological accuracy compared with experienced technicians using computer-aided software.	Comparative study
2025	Cao et al. (74)	To proposes a DL approach to automate comprehensive chart filing of bitewings	Mask DINO, SparseInst, and Mask R-CNN	CNN	Tooth segmentation and labeling, dental finding classification	Bitewings	Chart filing in dental practice	Mask DINO models exhibited high effectiveness for tooth segmentation and labeling.	Experimental study
2019	Tuzoff et al. (75)	To propose a novel solution based on CNNs to perform teeth detection and numbering automatically for panoramic radiographs.	A module based on Faster R-CNN	CNN	Teeth detection and teeth numbering	Panoramic radiographs	Electronic dental records	The performance of the proposed computer-aided diagnosis solution is comparable to the level of experts.	Experimental study
2022	Estai et al. (76)	To evaluate an automated detection system to detect and classify permanent teeth	A three-step procedure relying upon CNNs,	CNN	Teeth detection and numbering	Orthopantomogram (OPG) images	Automatic filing of dental charts	The resultant automated method achieved high performance for automated tooth detection and numbering from OPG images.	Experimental study
2024	Mercier et al. (77)	To assess the reliability, accuracy, and time consumption of AI-based software compared to a conventional digital cephalometric analysis method on 2D lateral cephalogram.	WebcephTm	CNN	Cephalometric landmarks	Lateral cephalometric radiograph	Orthodontic cephalometric analysis	The differences in accuracy between the conventional digital technique and two AI-based software alternatives were not clinically significant except for specific measurements. The semi-automatic AI-based software option was more accurate than the automatic one and faster than conventional tracing.	Experimental study
2023	Lee et al. (78)	To propose a fully automatic posteroanterior (PA) cephalometric landmark identification model and compare its accuracy and reliability with those of expert human examiners.	ResNet 18	CNN	Cephalometric landmark	Posteroanterior cephalometric images	Orthodontic Cephalometric analysis	Comparable with human examiners, the fully automatic PA cephalometric landmark identification model showed promising accuracy and reliability and can help clinicians perform cephalometric analysis more efficiently while saving time and effort.	Experimental study
2025	Elgarba et al. (79)	To validate an AI–driven tool for automated virtual implant placement by comparing its accuracy, implant dimension selection, time efficiency, and consistency with a human intelligence (HI)–based approach for single posterior tooth replacement.	The Relu Creator cloud-based platform	–	Virtual implant placement	CBCT and intraoral scanning (IOS)	Dental implant planning	Artificial intelligence is reliable for virtual implant placement in missing mandibular (pre)molars, producing clinically acceptable plans comparable to human experts while operating faster and much more consistently than implant clinicians.	Experimental study
2024	Adel et al. (80)	To assess the predictability of Invisalign SmileView for digital AI smile simulation in comparison to actual smile treatment outcomes, using various smile assessment parameters	Invisalign SmileView	“Invisalign Practice App”	Smile prediction	Pretreatment and post treatment intraoral and extraoral records	Smile aesthetics	More optimal lip lines, straighter smile arcs and more ideal tooth display were achieved in actual post treatment results in comparison to the initially predicted smiles.	Experimental study
2023	Chen et al. (81)	To compare the accuracy of dental implant placement by a vitro model experiment	THETA, Yizhimei	Robotic system	Implant placement	Partially edentulous jaws models	Dental implant	The implant positioning accuracy of the robotic system, especially the angular deviation was superior to that of the dynamic navigation system	Comparative study
2024	Wang et al. (82)	To compare the accuracy in edentulous implantation.	Yakebot, CAIS template	Robotic system	Implant placement	Edentulous patients	Dental implant	The accuracy of robotic system in edentulous implant placement was superior to that of the CAIS template	Comparative study
2024	Li et al. (83)	To investigate the accuracy of immediate anterior implantation using static computer-assisted implant surgery (s-CAIS) and robotic computer-assisted implant surgery (r-CAIS)	Remebot	Robotic system	Implant placement	Immediate anterior implant placement patients	Dental implant	The accuracy of immediate anterior implantation with r-CAIS was better than that with s-CAIS.	Comparative study
2022	Shen et al. (84)	To evaluate the effects of an at-home AI-assisted dental monitoring application on treatment outcomes in patients with periodontitis.	DENTAL MONITORING^®^ (DM)	–	Periodontal parameters	Periodontitis patients	Oral health counseling and monitoring	Using AI monitoring at home had a positive effect on treatment outcomes for patients with periodontitis.	–
2023	Snider et al. (85)	To evaluate the effectiveness in improving the patient’s oral hygiene during orthodontic treatment	Dental Monitoring™ (DM™)	Artificial Intelligence Driven Remote Monitoring Technology (AIDRM)technology	PI, MGI	Orthodontic patient	Oral health counseling and monitoring	The oral hygiene of orthodontic patients rapidly worsens over the first 3 months and plateaus after about 5 months of treatment. AIDRM by weekly DM scans and personalized active notifications may improve oral hygiene over time in orthodontic patients.	Experimental study
2025	Kim et al. (86)	To compare the clinical effectiveness, defined as improved oral hygiene measured by plaque reduction and halitosis control	ITT (Mombrush; XiuSolution)	Mombrush ProCare app	SHS, QHI, halitosis, oral microbiota	Participants	Oral health counseling and monitoring	ITTs enable better oral hygiene management than MTs through dental professional feedback.	Experimental study
2021	Sangalli et al. (87)	To evaluate the effectiveness remote digital monitoring during orthodontic treatment	Dental Monitoring^®^	DM app	PI, GI, and White Spot Lesions (WSL)	Orthodontic patients	Oral health counseling, tele-monitoring	Integration of a remote monitoring system during orthodontic treatment was effective in improving plaque control and reducing carious lesions onset.	Experimental study
2024	van Nistelrooijet al. (88)	To develop and evaluate a fully automated method for visualizing and measuring tooth wear progression using pairs of intraoral scans (IOSs)	An automated method	StratifiedTSegNet, ICP algorithm	Tooth wear progression	IOSs	Oral health monitoring	The proposed automated method for monitoring tooth wear progression was faster and not clinically significantly different in accuracy compared to a manual protocol for full-arch IOSs	Experimental study

### AI in oral health education and consultation

Oral health education is an initiative aimed at increasing public awareness of the significance of oral health. It seeks to inform individuals about maintaining proper oral hygiene, preventing oral diseases, and fostering a healthy lifestyle. This educational effort is a crucial element in advancing public health. By disseminating knowledge about oral health and advocating for good hygiene and dietary practices, it effectively prevents oral diseases and improves quality of life. The integration of AI in oral health education and consultation further enhances public awareness and behavioral change concerning oral health through personalized and interactive methods (89, 90). Utilizing tools such as virtual assistants, tailored education, online consultations, and health monitoring, AI not only improves individual oral health management skills but also offers more efficient and intelligent services in oral healthcare (91–93). The increasing implementation of AI in oral health education and consultation has significantly bolstered public awareness and management capabilities related to oral health.

### Oral health knowledge dissemination and virtual health assistants

Artificial intelligence is a remarkable tool that enhances engagement with oral health knowledge and promotes active participation in oral care. AI possesses the capability to generate oral health information by extracting content from various materials, such as videos and images, and by transforming textual information into visual formats, thus enriching oral health education. It can produce interactive learning modules, videos, animations, and games that improve public understanding and retention of oral health information, thereby increasing user engagement (24, 25). AI-supported gamified methods of oral health education provide dynamic and interactive learning experiences, leveraging principles of behavioral psychology to motivate patients and foster their involvement in oral care (26). A study comparing a basic oral health application with a gamified version found that both improved mothers’ knowledge and practices; however, the gamified application resulted in a more significant reduction in children’s plaque index, indicating superior clinical outcomes for gamified oral care (27). Furthermore, Virtual Reality (VR) allows individuals to experience an immersive and highly realistic three-dimensional (3D) virtual environment. This technology provides a range of sensory stimuli—visual, auditory, tactile, and olfactory—that enable users to vividly engage with a non-virtual reality. Both virtual reality and augmented reality technologies facilitate user interaction with virtual toothbrushes, dental floss, and mouthwash, thereby teaching correct dental care techniques. These immersive educational experiences not only reinforce good oral hygiene habits but also promote improved oral health outcomes by encouraging individuals to take an active role in their own dental care routines. By utilizing natural language processing (NLP) capabilities, AI can translate complex dental terminology into layman’s terms, making it more accessible for the general public (28). Additionally, AI can create tailored educational activities for specific demographics, such as the elderly and children, to disseminate oral health knowledge more effectively.

AI-powered virtual assistants and chatbots are software applications designed to simulate human conversations through the use of AI technology. These systems are programmed to understand and interpret user-input natural language, enabling them to handle text-based queries and commands. This allows users to engage in a fluid and conversational manner. Studies on the FunDee chatbot have shown that it yields remarkable effects in preventing dental caries, reducing dental plaque, improving feeding practices, increasing participation in toothbrushing, and enhancing oral health literacy, thus providing a viable alternative for promoting oral health (29, 30). AI-driven chatbots can interact with users across various platforms, including websites, mobile applications, and social media. They are capable of providing answers to general questions related to oral health and offering guidance on various aspects of oral hygiene, such as brushing, flossing, diet, and overall oral health management (31–34). Additionally, they assist users in establishing and maintaining optimal oral health habits. These virtual assistants are available around the clock to provide support and information, facilitating a more efficient and streamlined communication process. Users can upload photos or provide detailed descriptions of their symptoms through AI tools, allowing the virtual assistant to proactively analyze the data and offer relevant health advice or recommend seeking medical attention (35). AI-driven oral health self-monitoring systems, such as OHAI Advisors, utilize artificial intelligence technology and the selfie capabilities of contemporary smartphones to analyze images uploaded by users (94). These systems are designed to detect early signs of oral diseases, such as gingivitis, and assist users in self-monitoring their oral health (36). Following analysis, the system provides personalized oral care recommendations, aiding users in implementing timely preventive measures. Through continuous monitoring and feedback mechanisms established by this advanced technology, users are encouraged to adhere to optimal oral hygiene practices, thereby minimizing the risk of developing oral diseases (37, 38). This innovative AI technology empowers individuals to conduct comprehensive oral health assessments at their convenience and comfort in their own homes, facilitating early detection of potential issues and promoting timely intervention and treatment. AI technological advancement presents substantial potential for the early detection of oral diseases and contributes to the development of a proactive prevention strategy.

### Personalized oral health education content

The application of artificial intelligence (AI) allows for the efficient analysis of a wide range of user data, including health status, medical history, demographic information, and interaction patterns with educational materials (95, 96). This analysis enables AI to identify user preferences and specific needs, facilitating the creation of personalized oral health education materials. For instance, if a user indicates a preference for visual learning, AI can prioritize the delivery of educational videos or interactive simulations over text-based information. These materials may include tailored recommendations for nutrition, appropriate healthcare practices, and preventative measures for diseases, ultimately enhancing user health awareness and fostering self-management capabilities. Following a surgical tooth extraction, for example, AI can generate data pertaining to post-operative oral care, nutritional protocols, physical activity, and medication. Individuals often demonstrate increased attention to information that is most relevant to their personal circumstances. By providing personalized educational content that aligns with users’ unique needs, preferences, and learning styles, AI can enhance user engagement and comprehension. Furthermore, AI-driven oral health applications, leveraging user data inputs, can offer real-time feedback to assist users in modifying their oral hygiene practices (90, 94). AI also has the potential to deliver ongoing support and reminders to encourage the adoption and reinforcement of positive behaviors, as well as mitigate dental anxiety (39, 40). For example, through mobile and intelligent devices, AI can send users reminders related to oral hygiene practices, including tooth brushing, dental floss usage, and routine dental check-ups. These reminders are designed to encourage and promote effective oral hygiene practices among users, thereby reducing the risk of oral health issues. Users are empowered to document their oral health data, and an AI-driven system meticulously analyzes this information to produce detailed health reports and personalized improvement recommendations (41).

### Community group oral health interventions

Group oral health interventions aim to enhance the oral health of communities or specific populations through targeted programs, education, and preventive measures. These interventions focus on addressing prevalent oral health issues, raising awareness, and promoting healthier behaviors at the population level. AI can analyze community-level health data to identify risk factors and develop tailored public health programs (97). Additionally, AI can utilize digital technologies to monitor oral health trends across various populations, such as the elderly, pregnant women, and school-aged children, thereby facilitating better resource allocation and assessment of intervention effectiveness (42, 43). Personalized feedback and educational content can be delivered through mobile applications or other digital platforms, encouraging healthy oral hygiene habits within the broader context of community initiatives. Furthermore, AI can evaluate the effectiveness of different outreach strategies, optimizing the dissemination of educational content. Oral health is a critical component of overall health, and group oral health interventions represent a cost-effective strategy for enhancing societal health (98). By optimizing these interventions, AI can improve their efficiency and effectiveness, ensuring that advancements in oral health benefit the largest number of individuals possible.

### Training and supporting professionals

Training and supporting professionals is a crucial element in enhancing the effectiveness of population oral health interventions. By providing continuous training and support, we can ensure that professionals possess the necessary knowledge, skills, and tools to effectively manage oral health. Intelligent technology, particularly AI, can significantly enhance the efficiency and personalization of training while offering real-time support, enabling professionals to continually refine their skills and address complex tasks (13). AI can deliver ongoing educational resources for dentists and oral health workers, keeping them informed about the latest developments in oral health knowledge and treatment methods. In particular, professional AI models optimized for specific fields on the basis of general-purpose language models demonstrate greater advantages in educational and clinical applications (44). Additionally, AI can offer personalized training plans tailored to each professional’s knowledge level and learning needs, facilitating a more efficient mastery of essential oral health competencies. AI-powered virtual reality (VR) and augmented reality (AR) technologies can also create realistic simulation training environments for oral health workers (45, 99). By analyzing real cases using AI technology, professionals can enhance their understanding of patient needs and best practices in oral health management. AI can process vast amounts of data within the oral health field, including clinical cases, treatment outcomes, health survey data, and more, to generate detailed reports and trend predictions. This data analysis enables professionals to gain insights into current trends in oral health issues, risk factors, and effectiveness assessments, thereby optimizing training content and decision-making in real-world scenarios. Furthermore, AI-driven chatbots and virtual assistants can provide professionals with round-the-clock, real-time support (46). These virtual assistants can address inquiries related to oral health management, offer operational advice, and supply clinical reference materials. With the aid of AI technology, professionals can more efficiently acquire new knowledge, enhance their clinical skills, and effectively tackle complex issues in population oral health interventions, ultimately contributing to the achievement of public health goals.

### AI in early detection and diagnosis of oral diseases

Early diagnosis and intervention are regarded as the most critical stages in disease treatment. Deep learning (DL), convolutional neural networks (CNN), and data mining technologies, which assist in identifying patterns within data, can be effectively utilized in healthcare systems for the diagnosis, prediction, and classification of diseases (100). In comparison to traditional diagnostic methods, AI tools have the potential to enhance accuracy, reduce costs, and save time. By integrating AI with existing clinical images of oral diseases and training deep machine learning (DML) algorithms using multi-sample data, we can investigate the relationship between input images and corresponding diagnoses. This methodology facilitates the development of a multi-layer deep neural network model, capable of generating highly sensitive and specific techniques for disease recognition, thereby enabling precise screening and diagnosis of oral health issues. CNNs have demonstrated exceptional proficiency in the segmentation and recognition of dental features, proving to be a valuable asset for diagnosing various conditions, including maxillary sinusitis, detecting cephalometric landmarks, classifying root morphology, and determination of growth and development, among other applications utilizing panoramic X-rays (47–52). ChatGPT (OpenAI) is capable of generating radiological reports based on dental panoramic X-rays, but it currently still faces challenges regarding accuracy and reliability (53). By analyzing intraoral photographs, dental X-rays, CT scans, and other imaging data through deep learning algorithms, artificial intelligence has demonstrated high diagnostic accuracy for a variety of oral diseases, including caries (54–56), periodontal disease (57–59), endodontics (60, 101), periapical disease (61), oral mucosal diseases (63, 102), malocclusion (62), osteoporosis (64), and oral tumors (65). Generative models can be trained on extensive datasets of medical records and images, such as MRI and CT scans, to identify disease-associated patterns. For instance, Generative Adversarial Networks (GANs) have been employed for tasks such as image reconstruction, synthesis, segmentation, registration, and classification. Moreover, GANs can generate synthetic medical images that can be utilized to train machine learning models for image-based diagnosis or to augment existing medical datasets. Large Language Models (LLMs) represent powerful AI tools that have exhibited significant potential across various Natural Language Processing (NLP) tasks. LLMs can enhance the outputs of multiple computer-aided diagnosis (CAD) networks, including diagnostic networks, by summarizing and reorganizing information presented in natural language text formats. Investigating the characteristics and histopathological changes of potential malignant diseases that may precede Oral Squamous Cell Carcinoma (OSCC) will facilitate early diagnosis and help prevent disease progression. With the support of AI models, the diagnosis of high-risk oral cancer lesions can lead to improved patient survival rates. By leveraging machine learning models to analyze patients’ health data and medical histories, AI can predict the risk of future oral diseases, including oral cancer (66–68). This predictive capability facilitates early intervention, enabling healthcare providers to implement preventive measures before the disease advances. Such proactive strategies can significantly enhance patient outcomes by identifying individuals at high risk and offering customized treatments or regular monitoring, ultimately decreasing the incidence of severe oral health conditions.

### AI in dental disease diagnosis and treatment

The application of AI technology in the diagnosis and treatment of oral diseases seeks to assist dental professionals by enhancing clinical examinations, summarizing imaging and pathological data, and analyzing medical information through big data mining. This process entails the automatic identification of clinical variables and patient indicators, and is known as AI-assisted oral diagnosis and treatment. It signifies a substantial advancement in the integration of AI within clinical dentistry.

### Medical guidance and appointment scheduling

Artificial intelligence medical guidance and appointment scheduling are key applications of AI in modern healthcare, enhancing patient experience and streamlining clinical workflows (103, 104). AI-driven medical guidance uses natural language processing (NLP) and machine learning algorithms to interact with patients through chatbots or virtual assistants. Intelligent oral healthcare assistants can provide personalized and precise medical services based on the patient’s condition and information, and use facial recognition technology to identify patients, making processes like registration, consultation, payment, and appointment scheduling more convenient. This helps optimize the treatment process, reduce waiting times, improve consultation efficiency, and avoid wasting time. At the same time, AI can intelligently schedule appointments based on the patient’s oral health condition, properly arrange consultation times, and provide limited access to emergency oral care. It also optimizes the treatment process, maximizing the effective use of oral healthcare resources and minimizing resource waste. AI medical guidance and appointment scheduling not only optimize clinical workflows but also enhance the overall patient experience by providing personalized, efficient, and timely care, while reducing administrative burdens and improving resource allocation (69).

### Personalized treatment plans

Through the training on extensive patient datasets, AI can pinpoint patterns and correlations that human doctors may not instantly recognize, which can then be used to create personalized treatment plans tailored to the individual needs of patients (105). For instance, AI could potentially ascertain that patients with a certain genetic marker respond particularly well to a specific medication, and thus prioritize recommending this medication to patients with that genetic marker. Generative AI technology enables the analysis of a patient’s comprehensive medical history, including genetic data and lifestyle information, to accurately predict their potential responses to various treatment modalities, helping dental practitioners formulate tailored treatment strategies. Such precision-tailored plans are able to respond effectively to patients’ unique needs and can greatly enhance the effectiveness of treatment. Digital dentistry is rapidly advancing the development of precision medicine, and new data-driven diagnostic and treatment options will contribute significantly to the improvement of oral health (70, 71). Various digital technologies, including novel imaging techniques, 3D printing, robotics, and augmented reality/virtual reality (AR/VR), are guiding personalized diagnosis and treatment for patients, significantly optimizing their efficiency (72). The application of AI to automatically locate anatomical landmarks on cone-beam computed tomography (CBCT) images and classify dentofacial deformities can facilitate 3D diagnosis and virtual treatment outcomes (48, 51, 62). AI can also be utilized to optimize personalized orthodontic appliance design, precisely calculate the path of tooth movement and generate individualized treatment protocols with the objective to shorten therapeutic duration and enhance treatment outcomes. Intraoral scanning technology provides a comprehensive 3D digital recording of tooth and gum outlines. With the application of AI, this process facilitates the reconstruction of soft and hard tissue structures, contributing significantly to the success of anterior aesthetic restorations (72, 73). AI models based on 3D facial scans can automatically evaluate facial shape characteristics, which are used not only for diagnosis and planning in plastic and reconstructive surgery, but also to predict specific postoperative outcomes, thereby facilitating effective pre-operative communication with patients.

### AI dental diagnosis assistance systems and surgical robots

Artificial intelligence has been harnessed to devise automated software systems capable of streamlining dental diagnosis and data management, thereby facilitating and guiding dental professionals in making informed and beneficial decisions. The Artificial Intelligence Dental Diagnosis and Treatment Support System (also known as AI Dental Assistant or Dental Medical Assistant) is capable of facilitating the entire process of dental diagnosis and treatment within dental clinics (106, 107). It provides a highly efficient approach to managing both acute and chronic oral diseases by AI-based triage and graded diagnosis programs, interacting via handwritten or spoken communication. Additionally, it provides for automatic referral and consultation facilitated by AI, generates optimized examination plans based on medical orders, and furnishes precise diagnoses and treatment plans. The AI dental support system can significantly enhance the general quality of oral health care on a national level and fostering a balanced progression in this area. The implementation of standardized electronic medical records and pre-determined case templates can free physicians from the burden of medical documentation, allowing them more time for research into causes, diagnostic analysis, and treatment. Moreover, applications integrating large language models (LLMs) within electronic medical records can offer an enhanced layer of review for minimizing medical errors and capturing any overlooked information. For example, they can cross-reference current symptoms and diagnoses with past medical history to alert doctors to conditions that require further investigation, or scan medication lists to warn of potential adverse interactions or contraindications.

Generative AI models can assess and comprehend patient data, such as medical history, medications, allergies, and lab results, understand key points, and generate concise summaries highlighting critical information, such as diagnoses and recommended treatment plans. They can also identify trends in a patient’s health status over time. By automating this process, doctors can save time and ensure that no important details are overlooked. In addition, these clear and concise summaries of a patient’s health status can prove invaluable in enhancing communication between doctors and their patients as well as between different doctors involved in a patient’s care. AI models such as Convolutional Neural Networks (CNNs) and Artificial Neural Networks (ANNs) have demonstrated application potential in a variety of oral subspecialties, including auxiliary diagnosis, which has significantly improved clinical efficiency (74). For example, they have been used to study the anatomy of the root canal system, detect apical periodontitis and root fractures, determine working length measurements, predict the vitality of dental pulp stem cells, and forecast the success of retreatment procedures. The application of AI has proven to be accuracy in diagnosis and prognosis assessment in the field of dentistry. The use of AI can help improve treatment planning, which in turn can enhance the success rate of endodontic outcomes (108). Several studies have demonstrated that AI-driven CNN models for tooth identification exhibit high accuracy, comparable to expert-level performance (75, 76). AI technologies enable dentists to input their dental charts digitally, thereby improving efficiency. Cephalometric analysis is an essential process in orthodontics, yet the manual execution of this procedure is susceptible to human error. AI technology can identify cephalometric landmarks in 3D and 2D images, assisting clinicians in performing cephalometric analysis with higher precision and efficiency, thereby saving time and effort. However, further research is still needed to confirm the accuracy of artificial intelligence in cephalometric tracing (77, 78, 109). The oral and maxillofacial system, as a whole, requires that all treatments and recommendations be based on the accuracy of examination results and diagnosis. A comprehensive assessment of the patient’s teeth, muscles, and related tissues is necessary, to establish a correct diagnosis. Cone Beam Computed Tomography (CBCT), intraoral scanners, Computer-Aided Design/Computer-Aided Manufacturing (CAD/CAM), electronic facebows, and facial scanners can aid in the digital reconstruction of patient’s anatomic structures. The acquisition of detailed data on teeth, muscles, joints, and facial expressions has greatly facilitated the diagnosis and treatment in oral and maxillofacial rehabilitation, allowing for the creation of sustainable, aesthetically pleasing, and comfortable restorations that are well-suited to the oral and maxillofacial system. LMCD-OR is a large collection of dental X-ray images designed to support a wide range of AI-driven diagnostics (110). In implant prosthodontics, an intraoral detector can be used to identify the implant position and immediately transfer this data into the CAD program. AI has also improved the design and manufacturing of dental implants (70, 71, 79). AI technology is also used to predict treatment outcomes and patient responses, which is of great significance for clinicians in forecasting treatment prognosis (80).

The intelligent robotic surgical system (Da Vinci Surgical Robot), consisting of a console, a precise surgical robotic arm system, a clear imaging system, and minimally invasive surgical tools, has been successfully applied in the surgical treatment of numerous medical specialties. The use of surgical robots in oral and maxillofacial surgery has made the resection of deep facial tumors more precise and minimally invasive, achieving remarkable results (111–113). Some robotic solutions have already been commercialized, such as the “Yomi” robot (Neocis) for implant placement, especially in edentulous patients, and it has received FDA 510(k) clearance (114). The fully automated optical navigation system and dental implant robot developed by Professor Zhao’s team at the Air Force Medical University of China have also achieved the expected surgical outcomes. The research, development, and application of medical robots in various dental specialties, such as dental implantology, oral and maxillofacial surgery, prosthodontics, endodontics, and orthodontics, have also entered a period of rapid development (114, 115). The extensive skills and experience of skilled dental technicians and experienced dentists are integrated into the software of prosthodontic expert models. Subsequently, robots in prosthodontics are used to manufacture partial or complete dentures. Robotic technology is one of the many fields in medicine and dentistry that has rapidly advanced, driven by improvements in surgical planning, enhanced anatomical visualization, and haptic guidance during procedures—all of which help reduce human errors in patient care (81–83). Robotics has drastically altered the way clinicians think, as well as how they perform their duties. Simultaneously, it has enhanced the patient experience.

### Telemedicine

Thanks to AI technology, specifically communication tools like the internet, mobile devices, and video conferencing, tele-dentistry consultations are now feasible. Dentists can remotely diagnose, treat, and monitor patients through video conferencing and AI-assisted diagnostic tools, reducing the reliance on traditional in-person appointments (116). The rise of remote dentistry has made oral healthcare more accessible, especially for people in remote areas or patients with mobility issues. In addition to video consultations, patients are now able to utilize their smartphones to send medical information, such as high-quality intraoral photos, symptoms, or reports, to doctors, who can then review this information and respond with a diagnosis or treatment plan. Through wearable devices or oral health monitors, AI can continuously track patients’ oral health data in real time, thus promptly identifying any irregularities and alerting patients to take appropriate actions (84–86). Tele-dentistry services have increased the number of patients served, particularly during the pandemic, and have concurrently reduced costs, offering a reliable and effective alternative to traditional dental care services (87, 117).

### Dental monitoring and follow-up

Artificial intelligence can monitor and analyze patients’ oral health using advanced technologies such as image recognition and data analysis (37, 118). For instance, it can automatically evaluate the health of teeth, gums, and oral soft tissues through dental X-rays, CT scans, 3D imaging, and oral endoscopy (88). Additionally, AI facilitates real-time tracking and monitoring of patients’ oral health by integrating with dental health devices, including smartphone, smart toothbrushes and oral health monitoring instruments (37, 42). Furthermore, when providing dental care to elderly patients or those with multiple systemic conditions, AI technology automatically collects physiological indicators and vital signs, continuously monitoring and assessing their health status to better understand the function of various bodily systems. During procedures such as root canal treatments, extractions, implants, and oral maxillofacial surgeries, AI can provide real-time visual monitoring and intelligently predict overall health conditions, thereby supporting clinical decision-making and enhancing patient safety. The widespread adoption of wearable devices, such as smart bracelets and watches, allows for passive monitoring of various physical indicators in patients. These devices enable the collection and transmission of relevant health parameters, including psychophysiological signals, sleep quality, and physical activity, which can be utilized to monitor and manage stress before or after dental treatments (119).

Follow-up care after treatment is a critical component of oral healthcare; however, in clinical practice, it is often suboptimal due to limitations in manpower and resources. The integration of precise and intelligent AI robotic systems has facilitated the availability of AI medical assistants, which aid clinicians in conducting basic post-treatment Q&A with patients (24). These 24-h AI assistants can transcend time and geographical barriers, offering patients health education, preventive measures, and guidance for self-management (120). AI technology can automatically correlate various diagnostic and treatment data, comparing them with the patient’s annual oral health checkup results. Additionally, it can align and analyze imaging data from the same diagnostic indicators or sites at different times, enabling the detection of changes in key metrics and the calculation of disease progression. By employing continuous follow-up tracking through AI technology, clinicians can monitor disease outcomes and therapeutic effectiveness in real time, thereby enhancing follow-up rates. This approach is particularly beneficial for postoperative follow-up in treatments related to dental caries, periodontal disease, periapical diseases, oral and maxillofacial surgery, as well as long-term follow-up in dental restorations or orthodontic treatments. The application of AI in dental monitoring and follow-up significantly enhances diagnostic and treatment efficiency, accuracy, patient self-management capabilities, and the personalization and intelligence of oral health management.

### Improving efficiency and quality of healthcare services

Artificial intelligence can automate the management of patient appointments, medical records, and billing in dental clinics, thereby improving work efficiency and reducing the burden on healthcare staff (121). Furthermore, AI can enhance the quality of medical services and increase patient satisfaction. By analyzing patient flow and treatment demand, AI assists dental clinics in optimizing resource allocation and improving service quality (122). Additionally, AI has contributed to better organization of appointments, the provision of patients’ favorite music, and entertainment options, which can help alleviate anxiety symptoms and reduce stress levels. These improvements collectively enhance the overall patient experience and further increase patient satisfaction.

### Applications of AI in oral health data management

The advancement of artificial intelligence technology, coupled with the pervasive use of smart devices such as smartphones, has led to a transformation of the traditional single-source model of oral health data (123, 124). Individual users have emerged as key contributors to this data landscape. Contemporary oral health data encompasses not only conventional structured formats, such as numerical data and tables, but also unstructured records, including text, photographs, videos, and voice recordings. The management of this complex array of oral health data, along with its effective integration and expansion, will continue to enhance oral health prevention and care.

### AI in oral health data collection and analysis

**FIGURE 3:**
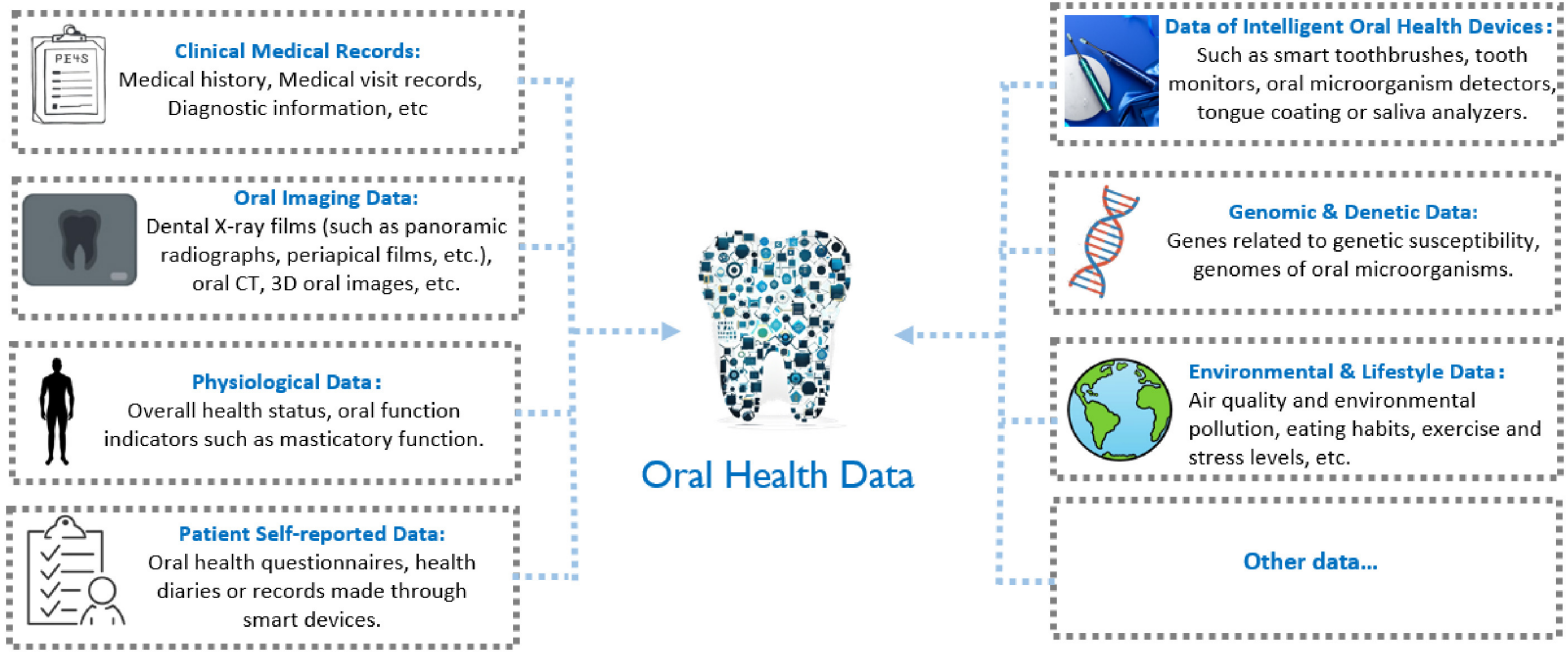
Sources of oral health data.

Oral health data is derived from a diverse array of sources, encompassing various aspects such as patient medical records, imaging exams, physiological information, and daily oral care behaviors (Figure 3). Clinical medical records serve as a crucial source of oral health data, incorporating the patient’s medical history, visit records, diagnostic information, treatment plans, surgical records, and prescriptions. Oral imaging exams are essential tools for evaluating oral health, including dental X-rays, oral CT scans, 3D oral images, and models, which provide detailed imaging data regarding teeth, gums, periodontal tissues, and other areas (125). Physiological data encompasses information about the patient’s overall health, indicators of oral function, and vital signs, enabling healthcare providers to conduct a comprehensive assessment of the patient’s health condition. Self-reported data from patients is primarily collected through questionnaires, health diaries, or recorded via smart devices. This self-assessment data may include information about their oral health status, descriptions of symptoms, and feedback on treatments. Intelligent oral health devices, such as smart toothbrushes, oral health monitoring instruments, and tongue or saliva analysis devices, can gather real-time data related to oral health. Certain oral diseases, such as periodontal disease and cavities, may be closely associated with an individual’s genetic factors. Consequently, genomic and genetic data are emerging as a significant area of focus in oral health research. Furthermore, environmental factors and lifestyle habits have a substantial impact on oral health, prompting the gradual inclusion of environmental monitoring and lifestyle data as integral components of oral health data (126).

Oral health data is derived from a diverse array of sources and encompasses various formats, including structured numerical data and tables, as well as a substantial volume of unstructured text records (127). This diversity complicates data collection and processing, necessitating thorough cleaning and preprocessing to ensure data quality and consistency (128). Such processes may involve tasks such as eliminating duplicates, addressing missing values, and normalizing the data. Effectively harnessing the integration and expansion of oral health data will continue to advance the prevention and management of oral health issues (123, 129). AI offers significant advantages in the realm of complex data processing due to its robust computational capabilities, automated data cleaning and preprocessing functions, precise pattern recognition and prediction abilities, and cross-domain data integration skills (130). As a pivotal technology within AI, machine learning excels at identifying patterns within large datasets and forecasting trends in previously unseen data. Deep learning models, which utilize neural networks with multiple hidden layers, can autonomously reveal intricate patterns within the data, enabling effective classification and prediction of complex features, with notable success in applications such as image recognition. Convolutional Neural Networks (CNNs), a fundamental architecture in deep learning, extract features from input data via convolutional layers and further refine and classify these features through multi-layered network structures. By providing data-driven insights and recommendations based on extensive datasets, AI can facilitate more efficient and effective decision-making, leading to improved outcomes and cost reductions (131, 132). The application of AI in analyzing oral health data offers valuable analytical support for the formulation of oral health guidelines.

## AI assessment and early warning systems

Artificial intelligence algorithms can continuously monitor various factors, including demographics, disease prevalence, and geographic distribution, to identify patients at higher risk for specific diseases, thereby facilitating prevention and treatment efforts. Artificial intelligence utilizes modeling, data mining, and machine learning techniques to analyze data encompassing medical history, demographics, and lifestyle factors. Predictive models can effectively identify patients at increased risk of developing these diseases and implement targeted interventions for their prevention or treatment (67, 68, 133). Furthermore, AI is capable of processing and analyzing large-scale oral health data, which assists researchers in studying oral health conditions and their associated risk factors. This capability aids in uncovering new disease patterns and risk factors, contributing not only to the development of an effective early-warning system for diseases but also encouraging individuals at risk to improve poor oral hygiene habits in their daily lives (134). Such improvements are crucial for reducing the incidence of oral diseases and advancing the field of oral medicine. In clinical trials, AI can be employed to analyze experimental data, evaluate the efficacy and safety of new treatments, and expedite the development of new drugs and technologies. An ideal healthcare model should begin with patients creating their own medical records during the pre-screening stage, which includes documenting their medical history, family history, and lifestyle habits. All examination results and images generated during treatment should be systematically recorded in a centralized database. This system enables AI to monitor examination results in real-time, compare follow-up results, track changes in oral health over time, and predict trends in oral health. If any negative factors are detected, the system will promptly notify both the patient and the physician for consultation. Additionally, data from various new mobile applications can facilitate diagnosis predictions, track progression or regression, and model individual patient characteristics over time. These applications are currently being integrated into clinical settings, utilizing web-based dashboards or tablets to remotely monitor participants’ symptoms and measurements in real-time. They provide alerts to healthcare providers when patients are at risk and evaluate medication adherence following dental or surgical treatments.

## Challenges of AI in oral health management

The impact of artificial intelligence on society has already been significant, and with advancements in technology, this impact is expected to grow even larger. However, there are still many challenges that need to be addressed.

### Technical and data challenges

The accuracy and completeness of AI-generated information are critical, as inaccuracies or omissions can lead to misdiagnosis or inappropriate treatment, potentially harming patients (135). It is essential to ensure that AI systems are meticulously designed and rigorously tested to yield reliable results. The training and predictive performance of AI models are heavily dependent on the quality and consistency of the data utilized. In essence, the intelligence and effectiveness of AI are determined by the data it receives. If a model is trained on incorrect or unreliable data, the results may be biased (106). Oral health data is often sourced from various origins, including electronic health records (EHR), imaging data, patient questionnaires, and clinical data from healthcare facilities (125). The format, standards, and quality of this data can vary significantly, which complicates data integration and processing. If the data contains missing, erroneous, or inconsistent information, the accuracy and reliability of the AI model will be compromised (136, 137). A machine executes algorithms that may be either programmed by humans or autonomously generated. However, flawed algorithms can yield biased outcomes. Data privacy and security remain pivotal concerns for the public and pose significant challenges for AI in the healthcare sector (138, 139).

### Clinical and implementation challenges

The data sources in oral health management are multifaceted, and there is a notable absence of unified standards regarding data formats, coding systems, and classification methods employed by various clinics, hospitals, and research institutions. This lack of standardization can result in difficulties in data integration, hinder the seamless sharing of information across different platforms and systems, and negatively impact the efficacy of AI models (139). AI is acknowledged for its ability to learn from extensive datasets, identify patterns, and make data-driven decisions. Although AI systems exhibit high efficiency in terms of speed and accuracy, they frequently fail to clarify the reasoning behind their conclusions. Given the complexity of AI technologies and their capacity for self-learning and behavioral adaptation, there remains a substantial gap in public understanding of AI. Consequently, AI encounters obstacles in areas such as updating dental knowledge and securing acceptance from both healthcare providers and patients (140, 141). Furthermore, a significant number of existing studies remain confined to retrospective trials, lacking prospective clinical trials and real-world effectiveness validation. Additionally, implementing such technologies may necessitate substantial initial investment, along with ongoing maintenance and repair costs (89). The intricate judgments required by clinicians during diagnosis and treatment, as well as in patient communication and ethical considerations, are domains that AI currently cannot fully navigate. How to seamlessly integrate AI tools into existing clinical workflows without increasing the workload of dentists, and ensure effective collaboration between AI and clinicians—leveraging the strengths of AI while avoiding over-reliance on it—poses another significant challenge.

### Ethical and regulatory challenges

Oral health data frequently encompasses sensitive personal information, posing significant challenges related to the protection of patient data, the prevention of data leakage and misuse, and the securing of informed consent for data usage. Furthermore, the integration of AI in decision-making raises ethical questions concerning accountability and the role of human judgment in patient care (142). When an AI diagnosis is incorrect, how to define the responsibility—should it be the responsibility of the doctor or the AI developer? In addition, the approval pathways, standards, and regulatory policies for AI software as a medical auxiliary tool remain to be explored and improved.

Despite the significant potential of artificial intelligence in oral health management, fully realizing its benefits requires overcoming a series of technical, ethical, and practical challenges. Effectively addressing these issues will enable broader applications of AI in the field of oral health and enhance overall oral health management.

## Future opportunities and development directions

Despite the aforementioned technical, clinical, ethical, and regulatory challenges, AI technology still holds broad prospects for transforming oral health management, with several key development directions emerging.

### Technological integration

As AI technology further matures and improves, its integration with intelligent digital devices for oral care will create new possibilities. For example, combining AI with oral intelligent digital devices (such as smart toothbrushes and intraoral scanners) enables real-time monitoring and analysis of oral health status (143, 144).

### Precision oral medicine

Artificial intelligence will drive the development of precision oral medicine, enabling personalized prevention, diagnosis, and treatment based on multi-omics data and individual lifestyle habits (95, 145, 146). Multi-omics data—including genomic, transcriptomic, and microbiomic data related to oral health—can be analyzed by AI models to identify genetic predispositions to oral diseases and microbial factors contributing to disease development. By integrating this data with individual lifestyle information (such as diet, smoking status, and oral hygiene habits), AI can develop personalized prevention plans and diagnostic models.

### Closed-loop oral health management

Artificial intelligence will play a key role in building an AI-empowered full-cycle closed loop of “screening-diagnosis-treatment-follow-up-health management” for oral health (147).

### Reducing healthcare costs and improving accessibility

Artificial intelligence technology, particularly with the popularization of communication tools such as the Internet, mobile devices, and video conferencing, has made tele-dental consultation and telemedicine possible. Through AI-assisted diagnosis and telemedicine, tele-dental services can increase the number of patients served while reducing costs (89, 93, 116).

## Discussion

Oral health education, monitoring, screening, diagnosis, treatment, and follow-up of oral diseases, along with the collection and management of oral health data, are fundamental components of oral health management. Artificial intelligence (AI) has shown significant advantages across various aspects of this field. Virtual oral health assistants have notably improved access to oral health education, while the early screening and diagnosis of oral diseases have emerged as prominent topics in current AI research (23, 90, 91). AI-assisted personalized treatment regimens have also been preliminarily attempted and yielded promising outcomes (146). AI has been adopted in all dental disciplines, including oral medicine, operative dentistry, pediatric dentistry, periodontology, orthodontics, oral and maxillofacial surgery and prosthodontics (148). Additionally, various dental sub-specialties have developed and launched specialized diagnostic and treatment robots (112, 115). The core functions of AI in oral health management encompass screening, diagnosis, evaluation, treatment, and follow-up of oral diseases. AI can enhance the accuracy and efficiency of these processes by leveraging large datasets, particularly through advanced imaging technologies such as CT scans, MRIs, ultrasounds, 3D facial scans, and endoscopic examinations, in conjunction with machine learning tools. While the applications of AI in diverse areas of oral health management is confronted with insufficient clinical validation and a variety of technical limitations, including inadequate sensitivity to early lesions and reliance on high-quality imaging data, artificial intelligence technology is expected to be further refined through practical clinical application experience and ultimately benefit patients. Looking forward, AI is poised to play an increasingly vital role in enhancing the efficiency and accuracy of oral health management. In conclusion, AI is bringing fundamental changes to oral health management. The application and regulatory experience of AI in other medical fields (such as radiology and pathology) can be drawn upon, as oral health management intersects with and shares partial similarities with these fields. However, the application of AI in oral health management encounters several limitations. Beyond the inherent challenges associated with the accuracy of AI technology when dealing with complex datasets, external factors also hinder its development. Effective machine learning algorithms require substantial amounts of patient privacy data, and the proper utilization and regulation of this information present significant challenges for AI advancement. The comprehensive development of AI systems not only requires close collaboration between oral clinical specialists and AI experts but also necessitates the joint participation of patients, policymakers, and enterprises to advance the application and development of AI in the field of oral health management.

## Limitations

This study has several limitations. First, the literature search was restricted to publications from 2019 onward. While this ensures the review reflects the most recent evidence and technological advancements, it may exclude seminal or foundational works published prior to this date. Second, a formal critical appraisal or reliability assessment (e.g., using tools such as the Cochrane Risk of Bias tool or Newcastle-Ottawa Scale) was not performed on the included studies. The synthesis therefore relies on the methodological rigor of the original sources as reported, and potential variations in study quality could influence the strength and generalizability of the conclusions drawn. While a formal critical appraisal of statistical methods in each included study was beyond the scope of this mapping review, our thematic analysis identifies recurrent statistical concerns—including issues related to sample size, validation, and reporting—as a key challenge that future primary studies must address to strengthen the evidence base. Future research would benefit from a broader temporal scope and a systematic quality assessment to further validate and contextualize these findings.

## Conclusion

Artificial intelligence has the potential to transform the prevention, diagnosis, and treatment models of oral health, but it is still in the early stage of transitioning from research to clinical practice. Future research should strive toward directions such as high-quality prospective clinical validation, addressing data and algorithm bottlenecks, the standardization of evaluation metrics and frameworks and establishing corresponding ethical and regulatory frameworks.
